# Ruptured Penetrating Atherosclerotic Ulcer Repaired With an Amplatzer Atrial Septal Occluder

**DOI:** 10.7759/cureus.37641

**Published:** 2023-04-16

**Authors:** Kyle J Clay, William F Campbell, Catherine E Lowe, Adam N Protos, Ashok Kumar Coimbatore Jeyakumar

**Affiliations:** 1 Department of Medicine, University of Mississippi Medical Center, Jackson, USA; 2 Division of Cardiovascular Diseases, University of Mississippi Medical Center, Jackson, USA; 3 Division of Cardiothoracic Surgery, University of Mississippi Medical Center, Jackson, USA

**Keywords:** penetrating aortic ulcer, amplatzer septal occluder, peripheral arterial diseases, heart tamponade, pseudoaneurysm, computed tomography, cardiovascular disease, aorta

## Abstract

Ascending aortic pseudoaneurysms are an infrequent but life-threatening complication of cardiac and aortic surgery. Although rare, these pseudoaneurysms can form as a complication of penetrating atherosclerotic ulcers. We report a case of a ruptured penetrating atherosclerotic ulcer repaired percutaneously with an Amplatzer Atrial Septal Occluder (Abbott, Plymouth, MN, USA).

## Introduction

Acute aortic syndrome includes acute dissection, intramural hematomas, and penetrating atherosclerotic ulcers. Penetrating atherosclerotic ulcers (PAUs) account for significantly fewer acute aortic syndrome presentations. However, the literature suggests that the rupture rate is higher than classic aortic dissections [1]. Rarely, PAUs can cause pseudoaneurysms. The incidence of this phenomenon is unclear. While this is more common in the descending aorta, it can also occur in the ascending aorta. Ascending aortic pseudoaneurysms (AAPs) are typically treated with surgical repair. However, these patients generally have extensive atherosclerotic disease burden and comorbidities that increase estimated surgical morbidity. With the introduction of percutaneous occluder devices, a handful of cases report using these devices to treat iatrogenic ascending aortic pseudoaneurysms [2,3]. We report a case of a ruptured penetrating atherosclerotic ulcer of the ascending aorta with pseudoaneurysm formation that was repaired percutaneously with an Amplatzer Atrial Septal Occluder (AASO; Abbott, Plymouth, MN, USA).

## Case presentation

**Figure 1:**
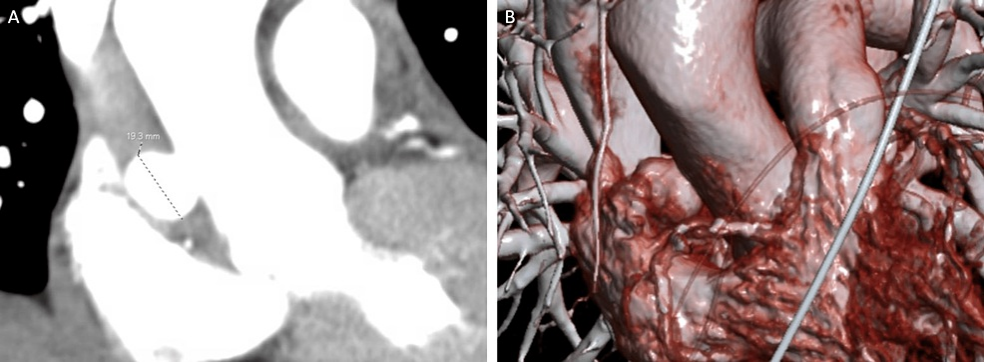
Initial Imaging. CT scan (A) and 3D reconstruction (B) of the chest showing a 1.9 x 1.8 x 1.7 cm proximal ascending aortic pseudoaneurysm.

A 55-year-old female presented after a syncopal episode. Her family reported that she collapsed on the ground at home. She was hypotensive upon arrival to our healthcare facility. Physical exam was significant for an ill-appearing female with cool, clammy skin, and 3+ pitting edema in her bilateral lower extremities. Her medical history was remarkable for hypertension, hyperlipidemia, fibromyalgia, chronic kidney disease, and a cerebrovascular accident with residual dysarthria and weakness. Differential diagnoses included seizure, cerebrovascular accident, transient ischemic attack, acute coronary syndrome, aortic dissection, and pulmonary embolism. An echocardiogram showed a normal ejection fraction and a large circumferential pericardial effusion with mildly elevated pericardial pressures by echocardiographic parameters. Computed Tomography (CT) scan with contrast showed a 1.9 x 1.8 x 1.7 cm right-laterally projecting pseudoaneurysm arising from the proximal ascending thoracic aorta just distal to the sinotubular junction and a periaortic hematoma compressing the ascending aorta (Figure 1). There was no active extravasation of contrast. It also showed atherosclerotic calcifications of the aortic arch, suggesting the etiology was a penetrating atherosclerotic ulcer. 

**Figure 2:**
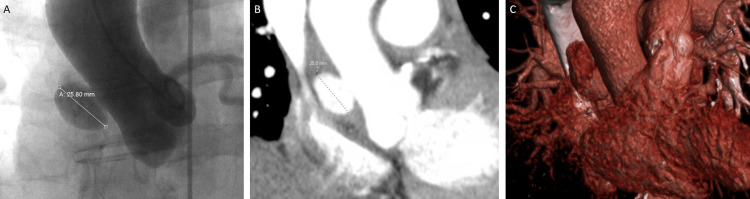
Follow-Up Imaging. Aortography (A), CT scan (B), and 3D reconstruction (C) of the chest showing an increase in the size of the ascending aortic pseudoaneurysm measuring 2.3 x 2.2 x 1.6 cm with new intramural extension of contrast.

She was started on intravenous vasopressor and inotropic support. Given her elevated pericardial pressures, she was aggressively fluid resuscitated, which allowed weaning of the vasopressors and inotropes. She was deemed a high-risk surgical candidate due to her multiple comorbidities and functional status. She was treated with aggressive medical therapy. Pericardiocentesis was not performed due to concern that the effusion was an extension of her PAU into the pericardial space. Two days later, follow-up imaging showed active extravasation and enlargement of the pseudoaneurysm to 2.3 x 2.2 x 1.6 cm (Figure 2). Her course was quickly complicated by renal failure, hepatic failure, severe thrombocytopenia, and coagulopathy. After a multidisciplinary discussion, the decision was made to undergo percutaneous intervention using an Amplatzer Atrial Septal Occluder.

**Figure 3:**
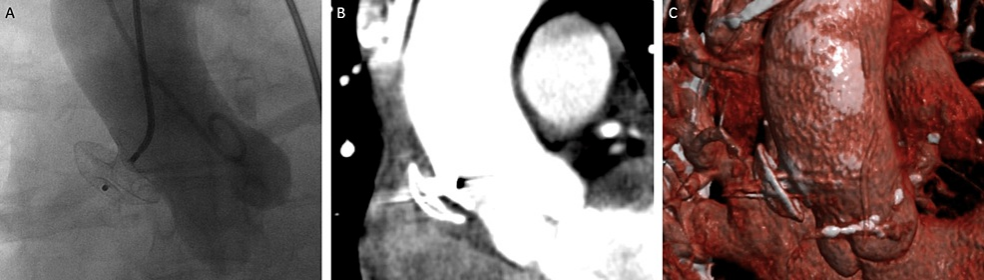
Post-Intervention Imaging. Aortography (A), CT scan (B), and 3D reconstruction (C) of the chest post-intervention showing no residual pseudoaneurysm or contrast extravasation.

Using a mini stick catheter and limited iliofemoral angiography to confirm the position of the arteriotomy, a 6F sheath was placed in the left common femoral artery and an 8F sheath in the right common femoral artery. Via the 6F sheath, a 6F angled pigtail was advanced over a wire to the aortic root. Ascending aortic angiography was obtained in a shallow right anterior oblique position, as this was the most perpendicular angle to the pseudoaneurysm neck by CT imaging. The pseudoaneurysm measured 26 mm in transverse diameter by aortography (Figure 2). An 8F AL1 guide catheter was advanced over a J-wire and used to engage the pseudoaneurysm. A 12 mm AASO was loaded onto the delivery cable of an Amplatzer Trevisio Intravascular Delivery System (Abbott). Although there was significant friction, the 12 mm AASO device was advanced through the 8F AL1 guide catheter. The AASO was deployed under fluoroscopic guidance. Prior to release, a repeat aortogram through the pigtail confirmed a stable position of the device, no obstruction to the right coronary ostium, and occlusion of the pseudoaneurysm (Figure 3). The 8F sheath vascular access site was closed with a Perclose Proglide (Abbott) suture and the 6F with a Vascade (Cardiva Medical, Santa Clara, CA, USA) without complication. Post-procedure imaging showed no residual pseudoaneurysm (Figure 3). She was restarted on her home antihypertensives, and her renal function improved. She recovered without incident and was discharged to inpatient rehabilitation. She was most recently seen nine months after the procedure and is without cardiac complaint. In addition, the device is in a stable position by echocardiography.

## Discussion

AAPs most often result from iatrogenic causes such as aortic or cardiac surgery at sites of aortic cannulation, aortotomy, or aortic anastomosis [2,3]. Less often, they form from trauma or infections. Although rare, these pseudoaneurysms can form as a complication of penetrating atherosclerotic ulcers. Stanson et al. defined PAUs as atheromatous lesions with ulceration that disrupt the internal elastic lamina and form a hematoma within the media layer [4]. PAUs account for significantly fewer acute aortic syndrome presentations. However, the literature suggests that the rupture rate is higher than in classic aortic dissections [1].

Most AAPs are treated with surgical repair with grafting, patching, suturing, or a Bentall procedure. However, these surgeries are often high-risk and involve repeat sternotomies, cardiopulmonary bypass, or hypothermic circulatory arrest. In one review, they carry increased mortality ranging between 6.7% and 41% [3]. When anatomy is favorable, endovascular techniques (e.g., grafting, coil embolization, and thrombin injections) have also been used to repair AAPs. Due to our patient’s comorbidities, she was deemed a prohibitively high-risk surgical candidate. Our patient's PAU likely ruptured, then a wall of fibrin, platelets, and clotting factors formed a pseudoaneurysm. During this initial rupture, she likely bled into the pericardial cavity and formed both a peri-aortic hematoma and an acute sanguineous pericardial effusion. As there was active bleeding and continued enlargement seen on follow-up imaging, conservative therapy was an unattractive option.

The Amplatzer Atrial Septal Occluder is a self-expandable device made of Nitinol mesh. Its structure consists of double discs connected by a waist. It was approved for use in 2002 by the FDA for secundum atrial septal defects. Bashir et al. described the first case of iatrogenic pseudoaneurysm repair by a septal occluder device [2]. Since then, there have been a handful of cases and a few case series [3,5]. The most extensive review by Quevado et al. summarized 36 cases of AAP closure with occluding devices [3]. All prior cases were pseudoaneurysms in patients with previous cardiovascular surgery. To the best of our knowledge, this is the first case of a ruptured penetrating atherosclerotic ulcer repaired percutaneously with an AASO device.

The AASO device is designed to be deployed through the Amplatzer Trevisio Delivery Sheath and Dilator. However, these are straight and require a significant amount of stiff wire past the deployment location to advance, which is not feasible or safe in this application. We used a large coronary guide catheter here to engage the pseudoaneurysm in a similar manner as a coronary ostium. Of note, AASO devices are sized by their central disc. The 12 mm AASO device that was used in this patient had a 26 mm distal (normally left atrial) and 22 mm proximal (normally right atrial) disc. Larger AASO devices require larger sheaths/delivery systems. This 12 mm AASO device is rated to be deployed through a 7F delivery sheath, but we were able to advance this, with difficulty, through the 8F guide, which is the largest guide we stock.

The Amplatzer Trevisio Intravascular Delivery System, introduced in 2021, was used in this case and offered a more flexible way to engage the pseudoaneurysm. The previous version, the TorqVue Delivery System (Abbott), has a much stiffer delivery cable, which would be more prone to pseudoaneurysm rupture. Also, device positioning is more difficult as the stiffer cable leads to more tension on the proximal disc while assessing correct placement. Potential risks include device malposition, migration, embolization, and failure to close the pseudoaneurysm completely [5,6]. However, with careful CT-guided pre-operative planning, these risks can be mitigated.

## Conclusions

Although PAUs are encountered less often than classic aortic dissections, they are still associated with significant morbidity and mortality. Since septal occluders were introduced, a handful of institutions report using them to repair iatrogenic pseudoaneurysms. With this case, we demonstrate the safety and efficacy of percutaneous closure of a ruptured penetrating atherosclerotic ulcer in the ascending aorta.
